# Morphology, Phase and Chemical Analysis of Leachate after Bioleaching Metals from Printed Circuit Boards

**DOI:** 10.3390/ma15134373

**Published:** 2022-06-21

**Authors:** Kamila Hyra, Paweł M. Nuckowski, Joanna Willner, Tomasz Suponik, Dawid Franke, Mirosława Pawlyta, Krzysztof Matus, Waldemar Kwaśny

**Affiliations:** 1 Department of Engineering Materials and Biomaterials, Faculty of Mechanical Engineering, Silesian University of Technology, 18A Konarskiego Street, 44-100 Gliwice, Poland; kamila.hyra@polsl.pl; 2 Materials Research Laboratory, Faculty of Mechanical Engineering, Silesian University of Technology, 18A Konarskiego Street, 44-100 Gliwice, Poland; miroslawa.pawlyta@polsl.pl (M.P.); krzysztof.matus@polsl.pl (K.M.); 3 Department of Metallurgy and Recycling, Faculty of Materials Engineering, Silesian University of Technology, 8 Krasińskiego Street, 40-019 Katowice, Poland; joanna.willner@polsl.pl; 4 Department of Geoengineering and Raw Materials Extraction, Faculty of Mining, Safety Engineering and Industrial Automation, Silesian University of Technology, 2 Akademicka Street, 44-100 Gliwice, Poland; dawid.franke@polsl.pl; 5 Department of Welding, Faculty of Mechanical Engineering, Silesian University of Technology, 18A Konarskiego Street, 44-100 Gliwice, Poland; waldemar.kwasny@polsl.pl

**Keywords:** metals recovery, recycling, bioleaching, scanning electron microscopy (SEM), high resolution transmission electron microscopy (S/TEM), X-ray diffraction (XRD)

## Abstract

The article presents the assessment of solutions and dried residues precipitated from solutions after the bioleaching process of Printed Circuit Boards (PCB) utilizing the *Acidithiobacillus ferrooxidans*. The obtained dried residues precipitated from bioleaching solution (leachate) and control solution were tested using morphology, phase, and chemical composition analysis, with particular emphasis on the assessment of crystalline and amorphous components. The analysis of the dried residues from leachate after bioleaching as well as those from the sterile control solution demonstrated a difference in the component oxidation—the leachate consisted of mainly amorphous spherical particles in diameter up to 200 nm, forming lacy aggregates. In the specimenform control solution larger particles (up to 500 nm) were observed with a hollow in the middle and crystalline outer part (probably Fe_2_O_3_, CuFeS_2_, and Cu_2_O). The X-ray diffraction phase analysis revealed that specimen obtained from leachate after bioleaching consisted mainly of an amorphous component and some content of Fe_2_O_3_ crystalline phase, while the dried residue from control solution showed more crystalline components. The share of the crystalline and amorphous components can be related to efficiency in dissolving metals during bioleaching. Obtained results of the investigation confirm the activity and participation of the *A. ferrooxidans* bacteria in the solubilization process of electro-waste components, with their visible degradation–acceleration of the reaction owing to a continuous regeneration of the leaching medium. The performed investigations allowed to characterize the specimen from leachate and showed that the application of complementary cross-check of the micro (SEM and S/TEM) and macro (ICP-OES and XRD) methods are of immense use for complete guidance assessment and obtained valuable data for the next stages of PCBs recycling.

## 1. Introduction

Year after year, a significant increase in the amount of produced waste of electrical and electronic equipment (WEEE) is recorded, and in 2014, its mass equaled 41.8 Mt (metric ton), in 2016—44.7 Mt, and 53.6 Mt was generated in 2019 [1,2,3]. In 2014, approximately 35% of electro-waste was recycled [4]. One of the electro-waste components is Printed Circuit Board (PCB). It is a laminate and constitutes about 3% of the mass of the whole electronic equipment [5,6,7]. A printed circuit board is manufactured from materials belonging to metals (about 30–40% of the whole component) and non-metals (plastics, ceramics, composites, about 60–70% of the whole component) [3,8,9,10]. It is possible to identify the diversified element composition: Cu, Zn, Ni, Al, Fe, Si, Ca, Pb, Sn, Cr, Mn, Mo, Ti, Pd, Pt, Ag, Au, etc. In some cases, a PCB consists of even as much as 60 elements, also toxic and hazardous [3,9,10,11,12,13]. Actions related to the recycling of WEEE and PCB protect the natural environment by limiting the use of ores, as well as reducing the amount of generated waste and pollutants (storage of heavy metals and other toxic substances). Additionally, recovery processes are less energy-consuming than the extraction of metals from primary sources [4,14]. Metal recycling from printed circuit boards is a complex issue, mostly due to the complicated construction of these components. The difficulty in developing effective methods of processing and recovery of those materials is connected mostly with the toxicity of some of the elements used to manufacture PCB, which is mentioned, among others, by Priya et al. [3] and Sohaili et al. [5], as well as a small concentration of metallic elements [3,5].

This paper is a continuation of research [15] aimed at the application of physical methods of metal recovery from PCB, which are economical and environmentally friendly. For this purpose, the material was prepared with a knife mill and in the next stage the fine grains were subjected to electrostatic separation. This method allows to separate grains into three products: concentrate (a mixture of metals), waste (mainly consisting of plastics and ceramics) for which two applications have been found, and a small amount of a difficult-to-manage intermediate (mixture of metals, plastics, and ceramics combined by strong connections). Due to its complicated structure and chemical composition, its processing with simple and cheap methods was difficult. Therefore, for this small amount of intermediate, it was decided to use bioleaching, which is also environmentally friendly, but its disadvantage is the long-term impact of micro-organisms on the components of PCBs during the catalysis of the metal recovery process. As a result of bioleaching, a metal-free sludge is to be formed, which will be combined with the waste (plastics) to produce composites and a solution containing metal ions that will be recovered in the next stage of the research [15]. It is a cost-effective process, and there are no required high energy inputs or advanced technology apparatus. Bioleaching with *Acidithiobacillus ferrooxidans* is a bacteria-assisted course (they provide and accelerate Fe ions oxidation), yielding solubilization of metals from material to leachate. Biological processes are inherently environment-friendly, but the kinetics are long-lasting. *A. ferrooxidans*, “iron bacteria”, are usually used both in laboratorial tests and industrial processes of bioleaching of copper, gold, nickel, or cobalt. They are chemolithoautotrophs, which live in the optimal temperature of 20–45 °C and pH = 1.3 ÷ 4.5. These bacteria take energy by oxidizing the reduced sulfur compounds (in the case of the oxidation of pyrite), but mostly from the oxidation of Fe^2+^ ions to Fe^3+^, thus ensuring continuous regeneration of the leaching agent, which is not possible in the case of traditional leaching [14,16,17,18,19,20,21,22,23,24]. The Fe^2+^ ions oxidize chemically with oxygen to Fe^3+^ ions for a very long time in a strongly acidic environment, but it is they who are responsible for the occurrence of the leaching reaction when bioleaching sparingly soluble sulfide minerals, whereas the presence of iron bacteria significantly accelerates the oxidation processes and Fe^3+^ regeneration with the pH value below 4, and only iron bacteria are capable of accelerating the oxidation with the indicated pH value. The process characterizes in high elasticity, due to the high adaptation ability of the microorganisms to extreme living conditions. The adaptation process, consisting in a gradual increase of the agent’s concentration in the bacterial environment makes it possible for the microorganisms to adapt to high metal concentrations. However, with a properly high concentration of heavy metals or some salts, the bacteria’s metabolism becomes disturbed, which may lead to their death [16]. In their study, Zhu et al. [17] emphasize also that plastics can contribute to bacteria’s faded activity during the bioleaching process, which, without the strain’s adaptation to such conditions, can also result in their necrobiosis. The presence of iron-oxidizing bacteria also increases the probability of the formation of secondary iron compounds (mainly jarosites), which are the undesirable products of bioleaching, as they form envelopes around the ore’s particles, thus inhibiting the kinetics of mineral solubilization [25]. In turn, solutions from bioleaching processes usually have very low pH values and high concentrations of sulfates and iron (III). Especially solubilized iron can disturb the further processes of separation and recovery of the metals [26,27]. After bioleaching, leachate (solid crystals of metals dispersed in liquid) and residue (non-metals and non-digested metals) are obtained. In the presented study, the leachate was the basis for metal recovery, hence the sludge has not been analyzed in detail.

The article analyzes the dried residues precipitated from solution after bioleaching using modern measurement techniques to evaluate and select applicable methods of recovering metals from solutions, such as e.g., the reduction of metal ions in the reactor in accordance with patent application No. P.410550 [28]. In order to properly design the process of recovering metals from a bio-solution, the input material for this process, in particular its chemical composition and morphology, should be identified. The aim of the study was to present possible and necessary analytical methods for testing solutions and the sequence of these analyzes. The intermediate in an amount less than 3% of total electrostatic separator feed (Suponik et al. [15]), which was subjected to the bioleaching process in this study, had a different chemical composition (especially in the amount of plastics) and structure from typical PCBs waste undergoing biological oxidation. 

Metal separation and recovery from solutions are commonly achieved by technologies such as solvent extraction (SX), electrolysis/electro-winning (EW), or ion exchange (IX). The effectiveness of these processes is diversified, and the range of results shows the influence of many factors on their course. Therefore, in order to apply and select the best method of recovering metals from solution obtained after bioleaching process of PCBs, these solutions must be fully characterized not only in terms of chemical composition but also crystalline and amorphous components should be assessed. This article was devoted to these issues. A secondary goal of this work was to demonstrate the possibility of using bacteria for effective leaching of PCBs waste materials.

## 2. Materials and Methods

This publication is a continuation of previous research presented in the paper Suponik et al. [15], in which the electrostatic separation of grinded PCBs was conducted. As a result, the following products were obtained: concentrate, intermediate, and waste, with yields of 26.2%, 2.8%, and 71.0%, respectively. The obtained concentrate and waste were pure and could be easily processed by known methods, unlike the intermediate, which consisted mostly of conglomerate grains (metal–non-metals–ceramics material). The release of metals from the conglomerate grains by mechanical means is time-consuming, costly, and difficult, mainly due to strong and undefined connections. Therefore, this material was biochemically processed with *A. ferrooxidans* bacteria in this study. A small amount of intermediate product (less than 3% after electrostatic separation) makes it possible to apply bioleaching as part of the recovery of metals from PCBs, which makes this long-lasting method effective and cheap compared with other methods.

The bioleaching was carried out in Erlenmeyer flasks (0.3 dm^3^) containing 3 g of the intermediate fraction samples and 0.18 dm^3^ of nutrient medium 9K (Silverman and Lundgren medium, composition in g/dm^3^: FeSO_4_·7 H_2_O—44.30, Ca(NO_3_)_2_—0.01, (NH4)_2_SO_4_—3.00, K_2_HPO_4_—0.50, KCl—0.10, MgSO_4_·7 H_2_O—0.50) inoculated by 0.02 dm^3^ of *A. ferrooixdans*. A pure strain of *A. ferrooxidans* (F3-02) was isolated from the source of mineral water coming from Głębokie (Nowy Sącz county, Poland) [29]. Metals concentration in the intermediate product of electrostatic separation (bioleached material) in %: Cu—6.68 ± 0.67, Al—1.34 ± 0.13, Pb—0.74 ± 0.07, Zn—0.74 ± 0.09, Ni—0.31 ± 0.03, Fe—1.5 ± 0.15, Sn—1.18 ± 0.12, Ti—0.39 ± 0.04 [25]. The bioleaching process was conducted for 64 days at ambient temperature, through systematic measurements of pH and Eh (every 3–5 days). To maintain the optimal solution pH, the samples were acidified (5M H_2_SO_4_) to pH = 2.0. After the end of the bioleaching, the solutions were filtered, by way of separating the remaining liquid with the use of medium filter papers (Macherey—Nagel, Allentown, PA, USA, MN 640 d, 18.5 cm ⌀) to separate the solutions and the residues from each other. Simultaneously, the sterile control samples were performed, under identical experiment conditions (chemical leaching).

For the pH and Eh measurements, a KnickPortamesstype 913 pH meter with an electrode by WTW pH—ElectrodeSenTix 41 with automatic temperature compensation (used to read off the liquid temperature) and an Elmetron CP—551 m with a Radelkis OP—7171—1A electrode were used, respectively. The specimens made from the solutions and the residue underwent further analyses—Inductively Coupled Plasma Optical Emission Spectrometry (ICP-OES), Ultra–high resolution Scanning-Transmission Electron Microscopy (S/TEM), Scanning Electron Microscopy (SEM), and X-Ray Diffraction (XRD).

The main analysis was carried out on the following specimens: made from filtered solution obtained after bioleaching process (described as leachate) and filtered solution obtained in chemical leaching (described as control solution). In order to perform SEM, S/TEM, and XRD analysis, dried residues precipitated from leachate samples and control solution samples were examined. To obtain representative results of electron microscopy analytical methods it was necessary to dilute the leachate and control solution with pure ethyl alcohol (1:1000) before the samples were dried. Without this procedure, it would be impossible to reveal the morphology of the particles placed in solutions, as well as obtain full information about the qualitative phase and chemical composition. Residue (sediment and solid phase filtered form leachate) from bioleaching process was tested only by using ICP-OES technique to show the chemical balance after the bioleaching process. 

A high-resolution scanning electron microscope (SEM) Zeiss Supra 35 (Carl Zeiss AG, Aalen, Germany) was equipped with the EDAX Energy dispersive X-ray spectroscopy (EDS) system (EDAX, Mahwah, NJ, USA) and enabled to analyze the chemical composition in micro-areas. The voltage accelerating the electron beam reached 15 kV. The solutions were mixed in a magnetic mixer for 15 min, then applied on a carbon band and dried at the temperature of 60 °C. The tests have been carried out on the diluted (morphology and chemical composition of selected areas) and undiluted solutions (only chemical composition).

In order to illustrate the morphology and structure of the examined specimens, a high resolution transmission electron microscope S/TEM TITAN 80-300 (FEI, Hillsboro, OR, USA) was used, the microscope was equipped with an X-ray energy dispersion spectrometer (EDS). The electron beam energy was 300 kV. For the analysis of the obtained results, the DigitalMicrograph by Gatan (v. 2.32.888), TEM Imaging & Analysis (v. 4.17) and Crystal Maker (v. 4.0) software was applied. To prepare the specimens, a proper number of the solutions was collected, which was then mixed in a magnetic mixer and applied in a small amount onto a copper mesh. In the EDS analyses, light elements were excluded (Z < 11), as their qualitative evaluation is burdened with too much error. 

The X-ray diffraction tests were made on an X’Pert Pro diffractometer (Panalytical, Almelo, The Netherlands), with the use of filtered X-ray lamp radiation (filter Fe) with a cobalt anode (KαCo λ = 1.7909 Å), powered by voltage 40 kV, with the filament current intensity = 30 mA. The examined specimens were applied on a non-reflective base made of silicon mono-crystal. The X-ray diffraction measurements were performed in the Bragg–Brentano geometry in the angular scope 10–70° [2θ] with the step 0.026° and the step count time 70 s. The obtained diffractograms were analyzed by means of the X’Pert High Score Plus software (v. 3.0e) with a dedicated Inorganic Crystal Structure Database—ICSD (FIZ, Karlsruhe, Germany).

In order to check and confirm the metals concentration in leachate and residue ICP-OES method was employed, using the Jy2000 spectrometer (by Horiba Yobin-Yvon, Hessen, Germany). The source of the induction was a plasma torch coupled with a 40.68 MHz frequency generator; the bioleaching products were previously dissolved.

## 3. Results

### 3.1. Bioleaching Process

**Figure 1:**
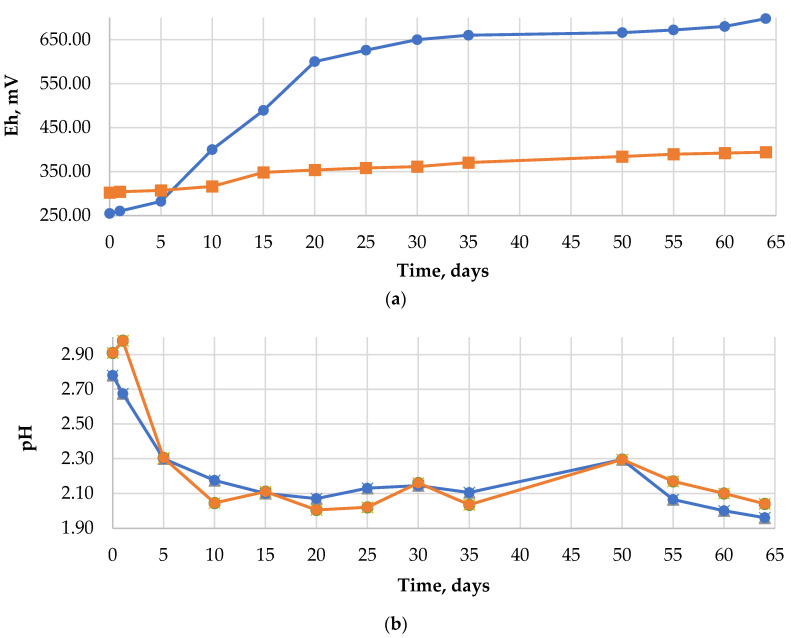
Graphs showing changes in: (**a**) redox potential (Eh), (**b**) pH, during bacterial (blue line) and chemical (orange line) leaching.

Figure 1 shows the changes in the potential of Eh and pH during bacterial and chemical leaching. For the system inoculated with the bacteria, a constant increase of Eh was observed—from the initial value of 255 mV to about 700 mV, obtained on the 52nd day of the experiment. The *A. ferrooxidans* bacteria gradually oxidized Fe (II) to Fe (III), which, in connection with a low pH during the bioleaching, points to growth of the microorganisms and proves a biological course of the reaction. At the same time, the value of Eh in the control solution remained in the scope of 300–400 mV. Due to the alkaline nature of the PCB waste [26,27] and to provide favorable conditions for microorganisms, the pH of the samples was corrected using 5M H_2_SO_4_. The reaction of the leaching solution inoculated with the bacteria was corrected several times in the preliminary phase of the process. The self-acidification effect with the maintained value of pH = 2 was observed on the 9th day of the performed process. In the case of the control solution, the pH correction was made regularly, thus ensuring an acidic environment during the 64 days of chemical leaching.

### 3.2. Scanning Electron Microscopy

**Figure 2:**
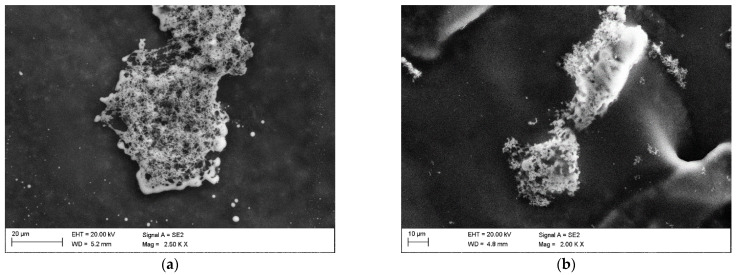
Morphology of the: (**a**) leachate, (**b**) control solution, SEM (SE).

The observations of the dried residues precipitated from solution after the bioleaching specimen made on a scanning electron microscope enabled an evaluation of the specimens’ morphology and chemical composition. Figure 2a,b shows the morphology of the dried residues precipitated from diluted specimen of the leachate and the control solution. The specimen obtained from leachate (Figure 2a) revealed mainly agglomerates with a spongy structure, probably formed as a result of evaporation of the liquid from the colloidal suspension. Similar structures, yet in a smaller content, were identified in the dried specimen from control solution. There was observed numerous agglomerates with a compact structure. 

**Figure 3:**
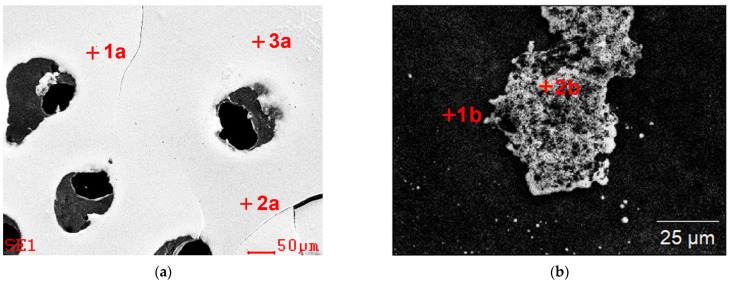

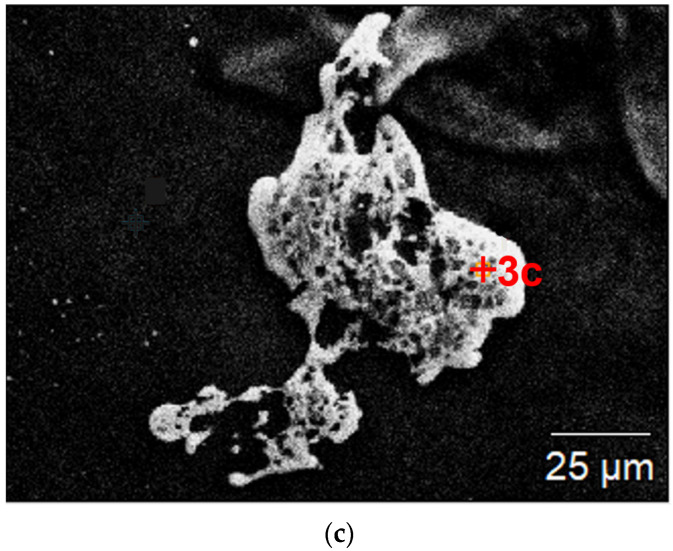
SEM images of the dried residues precipitated from leachate with marked chemical composition analysis points (results are presented in Table 1); (**a**) undiluted, (**b**,**c**) diluted.

**Figure 4:**
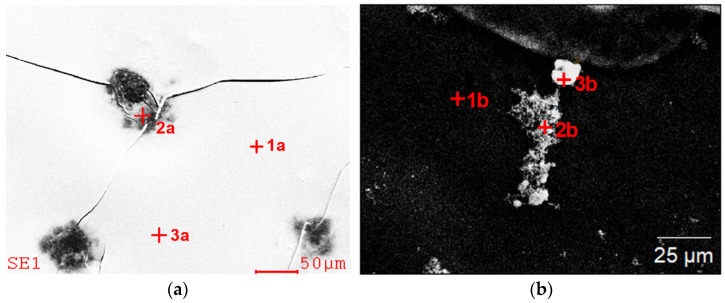
SEM images with marked chemical composition analysis points (results are presented in Table 2), dried specimen made from the control solution; (**a**) undiluted, (**b**) diluted.

The chemical analysis in the micro-areas of the dried residues precipitated from leachate (Figure 3 and Table 1) and control solution (Figure 4 and Table 2) revealed a content of the following elements: Cu, Fe, Al, Mo, Si, Ca, S. In the dried residues form control solution, Ag and K were also identified. A difference in the chemical composition between the formulation from the dried specimens from diluted solution and the undiluted one within the same test was observed as well. In the dried specimen from the solutions after the bioleaching, after dilution, no copper, magnesium or sulfur were identified, whereas in the specimen prepared from control solution, also silicon and potassium were not found, which were present in the specimen from undiluted solutions. This could be associated with the phenomenon of sedimentation of larger particles after the solution’s dilution.

### 3.3. High Resolution Transmission Electron Microscopy

**Figure 5:**
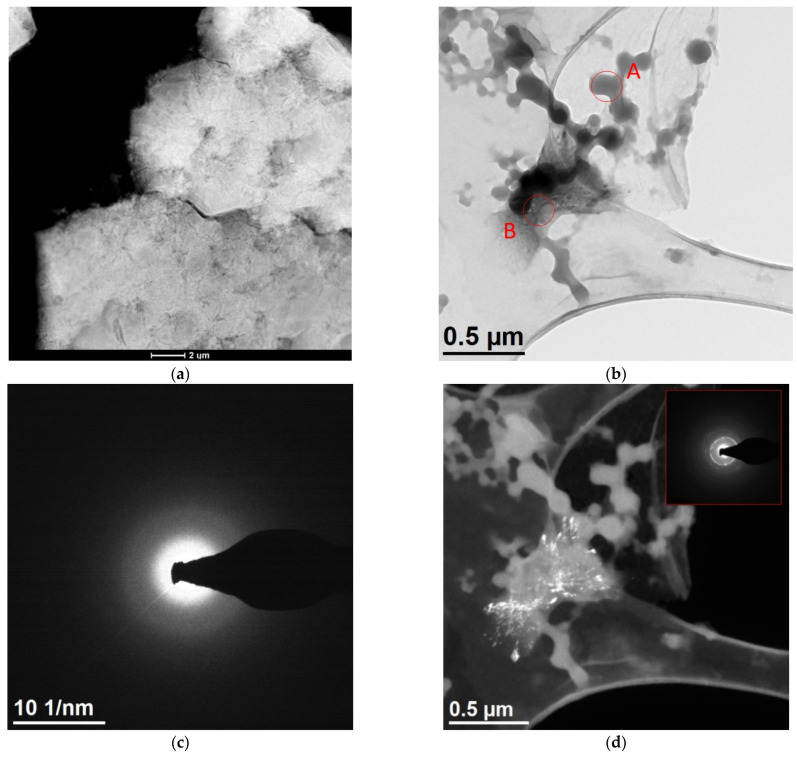
Microscope images of the dried residues precipitated from leachate; (**a**) undiluted (STEM HAADF—High-angle annular dark-field scanning transmission electron microscopy), (**b**) diluted (TEM), (**c**) selected area diffraction SAED obtained for area indicated by A image (**b**), (**d**) dark field image (TEM-DF), in right bottom part selected area electron diffraction from the area indicated by B in image (**b**).

**Figure 6:**
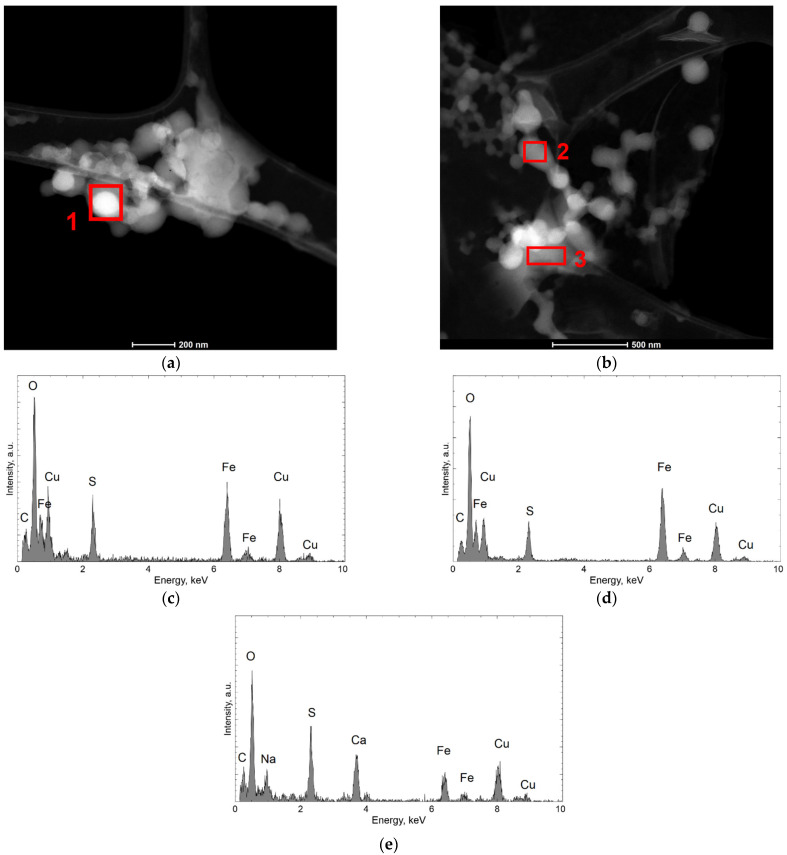
STEM-HAADF images of the dried residues precipitated from leachate, diluted; (**a**,**b**) spherical particle where EDS analysis were performed, (**c**) EDS result obtained in area indicated by 1 in (**a**), (**d**) EDS result obtained in area indicated by 2 in (**b**), (**e**) EDS result obtained in area indicated by 3 in (**b**).

Figure 5 shows the results of TEM observations of the dried residues precipitated from the solution obtained in the bioleaching process. The specimen from undiluted solution (Figure 5a) are characterized by a spongy structure. More precise analyses could be performed on dried specimen obtained from solution after dilution. Figure 5b shows particles of spherical shape, with a diameter of several tens to 200 nm, forming lacy aggregates. The diffraction made in this area (the example is marked by A in Figure 5b) confirms an amorphous structure (Figure 5c). Additionally, there are visible fragments with a different morphology (marked by B). Both the SAED (Selected Area Electron Diffraction) and the dark field image confirm the nanocrystalline structure of these fragments (Figure 5d). The analyses of the chemical composition (Figure 6a–e) obtained in STEM mode with the use of EDS confirmed that the aggregates made of spherical particles contain mainly Fe, S, and O (the presence of Cu in the recorded spectra may be omitted), while the irregular, nanocrystalline fragments contain additional Ca and Na. 

**Figure 7:**
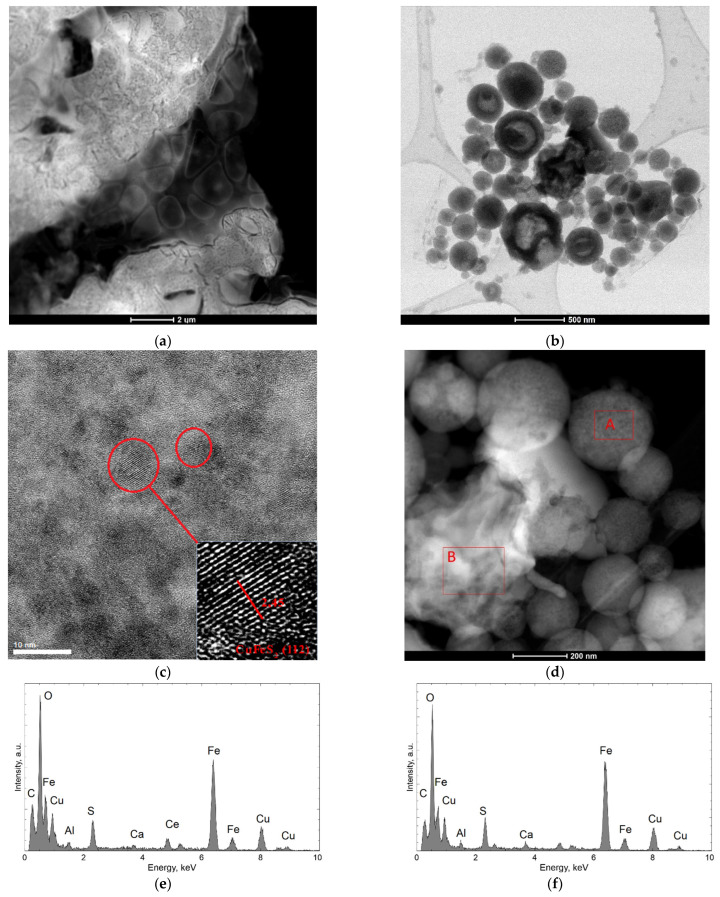
Microscope images of the dried residues precipitated from control solution; (**a**) undiluted (STEM HAADF), (**b**) diluted (STEM B), (**c**) TEM image of the outer part of bigger spherical particle from (**b**), (**d**) STEM-HAADF image consist of spherical and unregular fragments, where EDS analysis were performed, (**e**) EDS result obtained in area indicated by A in (**d**); (**f**) EDS result obtained in area indicated by B in (**d**).

Figure 7a–d shows the structure of dried residues precipitated from the control solution. A representative result of the specimen obtained from diluted solution is shown in Figure 7b. It shows spherical particles of various diameters (several dozen to 500 nm), which are not connected. Larger particles are hollow in the middle, while the outer part is crystalline (Figure 7c), which can prove incomplete oxidation. Spherical particles consist of Fe, S, and O. The presence of Ce is also visible in the spectra, which results from its presence in the PCB. Between the spherical particles, there are visible fragments with an irregular structure (marked as B in Figure 7d), which chemical composition that is similar to the spherical particles (Figure 7f).

### 3.4. X-ray Qualitative Phase Analysis

**Figure 8:**
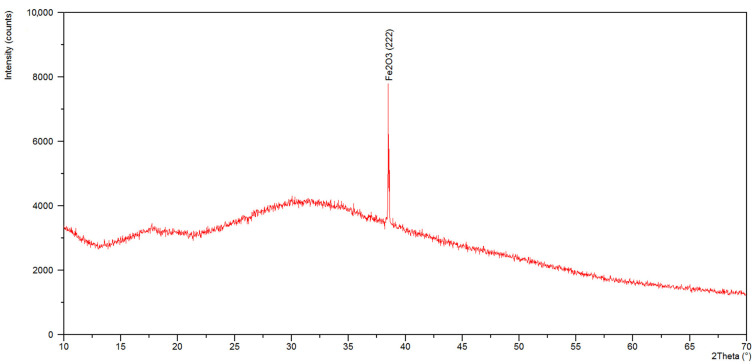
X-ray diffraction patterns of the leachate.

**Figure 9:**
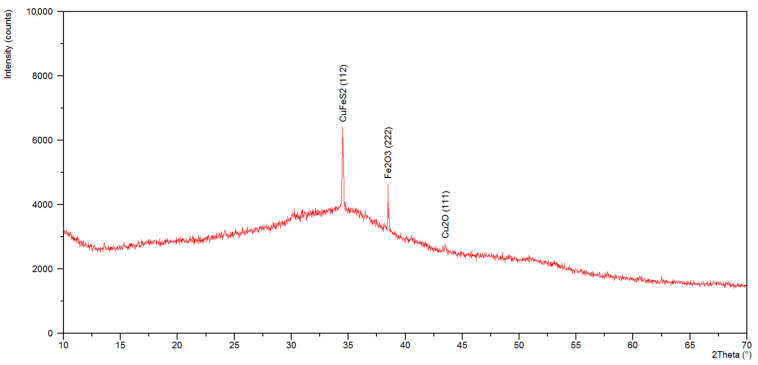
X-ray diffraction patterns of the control solution.

In the diffractogram of the specimen from the dried residues precipitated from solution after bioleaching (Figure 8) in the scope from 22 to 45° 2 Theta, we can see a very big, slightly asymmetric hump, characteristic of the amorphous component. A diffraction line in an angular orientation was also recorded, which corresponded to reflection from the crystalline planes (222) of iron oxide (III)—Fe_2_O_3_ (ICSD: 98-010-8905). The qualitative X-ray phase analysis of formulation made from dried residues from the control solution (Figure 9) revealed an amorphous component as well as diffraction lines which can be attributed to the strongest lines of the standard CuFeS_2_ (ICSD: 98-003-0289), Fe_2_O_3_ (ICSD: 98-010-8905), Cu_2_O (ICSD: 98-017-3983). In the case of the examined specimens, diffraction lines are characterized by low intensity, due to high content of an amorphous component.

### 3.5. ICP-OES Analysis

The chemical composition of leachate and residue is presented in Table 3. The theoretical maximum content of elements can be calculated in accordance with metals composition in the intermediate product of electrostatic separation which was later bioleached. The calculated theoretical maximum content of Cu, Al, Pb, Zn, Ni, and Sn was 1000, 200, 110, 140, 50, and 180 in ppm respectively. It could be observed that in the case of copper, aluminum, zinc, and nickel, the larger quantity of each substance appears in the leachate. Lixiviation did not occur in all other cases (lead and tin), where a bigger amount of elements was recognized in the residue. As regards copper, the bioleaching process was the most efficient, subsequently: zinc, nickel, and aluminum.

## 4. Discussion

The tendency for a change in the level of oxidation-reduction potential and pH in time (Figure 1) is in agreement with the studies published so far (Willner et al. [30]). During the bioleaching procedure, the microorganisms were provided with the optimal growth conditions; however, the experiment time was longer than it had been assumed. Probably, the waste composition—a ground PCB fraction rich in metals and an unseparated metallic fraction—had a slowing effect on the metabolism of the bacterial cells. The plastics present in the sample could have extended the time of the bacteria’s adaptation to the environment, which is mentioned by Zhu et al. [17]. 

Referring to the composition of metallic elements of the initial material (chemical composition of ground PCB after the separation of the plastic fraction (Franke et al.) [12]) as well as the solutions after the bioleaching, it can be stated that the iron percentage in the specimen increased significantly, which is related to the course of the leaching process and continuous oxidation of Fe^2+^ to Fe^3+^ guaranteeing a transition of the metal from the solubilized material into the solution. Analyzing the obtained SEM and TEM results, we can assume that the demonstrated small differences in the chemical composition between the obtained dried specimen from the undiluted solution and that from the diluted one were mainly connected with the applied research technique (analysis in micro-areas) as well as the preparatory procedure. The concentration of some elements in the formulation was low, and, with heavier/bigger agglomerates in the specimen made from undiluted solution, sedimentation could have taken place. However, the obtained results of the chemical composition confirm the presence of elements used to manufacture PCB in the solution, which is mentioned by Seif El-Nasr et al. [9], Liang et al. [10], Szałatkiewicz [11] van Houwelingen [13], and de Andrade et al. [31]. 

Bioleaching with *A. ferrooxidans* bacteria provides acquiring elements such as copper, zinc, and aluminum from leached material (Willner et al. [16], Kremser et al. [32], Rouchalova et al. [33]). It was confirmed in the ICP-OES results in the solution samples. Moreover, it was proved by SEM EDS and TEM EDS analysis results—in the dried residues precipitated from solutions the following elements have been identified: Cu, Fe, Al, Mo, Si, Ag, K, S, Ca, and Na. In the examined specimen from solutions, appearance of Pb and Sn has not been supported as opposed to residue (ICP-OES analysis findings). Similar observations were presented in previous papers by Brandl et al. [34], Ilyas et al. [35], and others—Bryan et al. [36], Willner et al. [30], Willner et al. [37], Hubau et al. [38]. It was reported that during PCB bioleaching, Sn was detected in residues in the form of precipitated SnO together with Pb in the form of PbSO_4_. Additionally, performed analysis results validate that a strong combination of metal–nonmetal–ceramics conglomerate were broken and elements from conglomerate probably entered the solution. The metal content in the leachate (Table 3) is sufficient for the reduction and precipitation of metal ions from the solutions using e.g., iron reactor (patent application No. P.410550, [28]), which is planned to be carried out in the second stage of the research.

TEM observations show that the morphology of dried residues precipitated from leachate after dilution consisted of mainly amorphous spherical particles in diameter up to 200 nm, forming lacy aggregates. Chemical composition confirmed that these aggregates contain mainly Fe, S, and O. In the dried specimen from control solution larger particles (up to 500 nm) were observed with hollow in the middle and crystalline outer part. The obtained investigation allowed the conclusion that the differences in the morphology of these spherical particles observed in the dried specimen made from leachate and the control solution are the result of activity of the bacteria and their participation in the solubilization of the waste components, with their visible degradation–acceleration of the reaction owing to a continuous regeneration of the leaching agent (Cui et al. [39]). Also, Arshadi et al. [40] point to the fact that the factor of the change in the morphology of the spherical particles are the bacterial metabolites, which, through their chemical operation, intensify the process of their degradation. 

Qualitative X-ray phase analysis of ground PCBs performed by Erust et al. [41] and Franke et al. [12] made it possible to identify mainly metallic phases (iron and copper). The investigation results presented in this article point to the presence of mostly oxides and sulfides of these metals in dried residues precipitated from solution, which is a consequence of bioleaching. The specimen made from leachate is characterized by a spongy structure, composed of spherical particles of iron oxide III, exhibiting features of an amorphous substance, which was confirmed by XRD. The specimen made from control solution, in turn, demonstrated the presence of Fe_2_O_3_ as well as a large content of fine-dispersive crystals, probably CuFeS_2_ and Cu_2_O, in the base of the amorphous phase. It can be assumed that these compounds were formed during the leaching process, with participation of elements present in nutrient medium, which also was confirmed by the study of Sethurajan et al. [8].

The application of such a variety of test methods has enabled comprehensive information on the leachates. Each of the analyses complemented each other and gave different data on the specimen. Neither could be omitted—without SEM it is impossible to perform XRD data, and without XRD it is difficult to identify the phase composition only by TEM analysis. The only difficulty in conducting the tests was the preparation of specimens which must be dried under the same conditions. It required a lot of attempts to achieve the intended goal—as indicated in the presentation of the results, it was necessary to dilute the solution to reveal the morphology of the particles in dried specimen (SEM and TEM), or multistage evaporation of water from leachate to prepare the specimen for XRD testing. The chemical composition analysis was difficult in terms of identifying some heavy elements that could fall to the bottom of the vial immediately after the mixing process was completed. Therefore, it took several attempts to collect test materials to obtain clear results. However, the obvious advantage of combining the above-mentioned research methods is the simultaneous obtaining of information about the material on a micro and macro scale. Previous publications on bioleaching have not used all the methods indicated in this study, most often it was confined only to the analysis of the chemical composition. This concerns in particular the intermediate obtained from electrostatic separation [28], which had different chemical composition and structure from typical PCBs. The use of electron microscopy made it possible to visualize the morphology of the specimen, which is interesting information and could potentially be used to define bioleaching mechanisms that are not yet explicitly defined. All the data obtained from the investigation using presented analytical techniques allow to assess the applicability of the best method of recovering metals from bio-leachate and to select the best one. This study will be carried out in the next step of the research.

## 5. Conclusions

The chemical composition of the leachate is similar to the composition of the elements used to manufacture PCB boards (Cu, Al, Mo, Ca, Ag, Mg, Si). The dominating amount of iron is involved with the continuous oxidation of Fe^2+^ to Fe^3+^, which ensures the transition of the metals from the solubilized material into the solution. According to copper the bioleaching process was the most effective.

The application of comprehensive scientific techniques allowed to evaluate the morphology of the obtained products and identify the components of the analyzed solutions, mainly the oxide and sulfide phases (Fe_2_O_3_, Cu_2_O, CuFeS_2_), which were presented as a consequence of the leaching processes. These complementary methods allow for a quick and complex analysis that gives full information about the analyzed solutions (ICP-OES analysis) and dried residues precipitated from leachate and control solution (SEM, S/TEM, and XRD), which is needed in the next step of PCBs recycling to conduct a qualitative and quantitative assessment of precipitates, obtained as a result of metal recovery from leachates by e.g., reduction of ion metals using an iron reactor.

The morphology of the dried residues precipitated from leachate after bioleaching consisted of mainly amorphous spherical particles in diameter up to 200 nm, forming lacy aggregates. In the dried residues precipitated from the control solution larger particles (up to 500 nm) were observed with hollow in the middle and crystalline outer part. The obtained investigation allows to conclude that the differences in morphology and phase composition of these spherical particles observed in specimen obtained from the leachate and the control solution are the result of activity of *A. ferrooxidans* bacteria and can be related to efficiency in dissolving metals during the bioleaching.

Obtained results of the investigation confirm the activity and participation of the *A. ferrooxidans* bacteria in the solubilization process of electro-waste components, with their visible degradation–acceleration of the reaction owing to a continuous regeneration of the leaching medium.

Electron microscopy was useful to assess the chemical composition and to obtain the images of solutions morphology, in order to examine the input and effectiveness of metal–nonmetal–ceramics conglomerates bioleaching. However, in a full assessment of the bioleaching process, complementary cross-checks of the micro (SEM and S/TEM) and macro (ICP-OES and XRD) methods are required.

## Figures and Tables

**Table 1:** Results of chemical composition analysis of the dried residues precipitated from leachate (EDS, SEM) at points shown in Figure 3; specimen from undiluted solution (Figure 3a); specimen from diluted solution (Figure 3b,c).

Element	Dried Residues Precipitated from Undiluted Solution (Figure 3a)						Dried Residues Precipitated from Diluted Solution (Figure 3b,c)					
	Point of Analysis											
	1a		2a		3a		1b		2b		3c	
	% wt.	% at.	% wt.	% at.	% wt.	% at.	% wt.	% at.	% wt.	% at.	% wt.	% at.
Cu	4	3	4	3	2	-	-	-	-	-	-	-
Fe	40	29	39	26	40	26	-	-	32	40	25	29
Al	2	3	2	3	2	3	-	-	-	-	4	14
Mo	-	-	-	-	-	-	-	-	64	60	64	43
Si	-	-	-	-	-	-	-	-	-	-	4	-
Ca	2	3	2	3	2	3	-	-	5	-	4	14
S	53	63	54	65	55	68	-	-	-	-	-	-

**Table 2:** Results of chemical composition analysis of the dried residues precipitated from control solution (EDS, SEM) at points shown in Figure 4; specimen from undiluted solution (Figure 4a), specimen from diluted solution (Figure 4b).

Element	Dried Residues Precipitated from Undiluted Solution (Figure 4a)						Dried Residues Precipitated from Diluted Solution (Figure 4b)					
	Point of Analysis											
	1a		2a		3a		1b		2b		3b	
	% wt.	% at.	% wt.	% at.	% wt.	% at.	% wt.	% at.	% wt.	% at.	% wt.	% at.
Cu	5	3	7	5	5	3	-	-	-	-	-	-
Fe	46	34	52	45	48	35	-	-	33	42	35	44
Al	2	3	2	-	2	3	-	-	3	8	2	6
Mo	-	-	-	-	-	-	-	-	62	50	59	44
Ag	-	-	-	-	-	-	-	-	3	-	2	-
Ca	-	-	-	-	-	-	-	-	-	-	2	6
K	2	3	2	-	2	3	-	-	-	-	-	-
S	46	58	36	50	44	58	-	-	-	-	-	-

**Table 3:** The chemical composition of the leachate and the residue (ICP-OES analysis).

Element	Quantity of the Substance /Element in the Leachate (ICP)	Quantity of the Substance /Element in the Residue (ICP)
	ppm	ppm
Cu	700	250
Al	150	60
Pb	20	70
Zn	75	60
Ni	34	20
Sn	10	200

## Data Availability

Not applicable.
